# Editorial: Functional and quantitative imaging of the lung

**DOI:** 10.3389/fmed.2024.1515096

**Published:** 2024-12-03

**Authors:** Mark O. Wielpütz, Jim M. Wild, Edwin J. R. van Beek

**Affiliations:** ^1^Subdivision of Pulmonary Imaging, Department of Diagnostic and Interventional Radiology, University Hospital Heidelberg, Heidelberg, Germany; ^2^Translational Lung Research Center Heidelberg (TLRC), German Center for Lung Research (DZL), Heidelberg, Germany; ^3^Department of Diagnostic and Interventional Radiology with Nuclear Medicine, Thoraxklinik at University Hospital Heidelberg, Heidelberg, Germany; ^4^Pulmonary, Lung and Respiratory Imaging Sheffield (POLARIS), Division of Clinical Medicine, Faculty of Health, School of Medicine and Population Health, University of Sheffield, Sheffield, United Kingdom; ^5^Insigneo Institute, University of Sheffield, Sheffield, United Kingdom; ^6^Edinburgh Imaging, Queens Medical Research Institute, University of Edinburgh, Edinburgh, United Kingdom

**Keywords:** functional imaging, quantitative imaging, lung, computed tomography, magnetic resonance imaging

Pulmonary functional imaging provides important functional and quantitative metrics for a wide range of pathological conditions. Functional lung imaging research is based on two main paradigms: (1) in the lungs, structural alterations cause inevitable loss in lung function, ultimately leading to perfusion and ventilation abnormalities and subsequent reduction of blood oxygenation. Several different structural compartments, such as the airways, conducting pulmonary arterial and bronchial arterial as well as venous vessels, the capillaries, and the tissue barrier, allowing diffusion of oxygen from the alveoli to the red blood cells, can be affected in various combinations. (2) Traditional pulmonary function testing such as spirometry and full body plethysmography is insensitive to early lung disease since healthy areas may compensate for inhomogeneously distributed function loss. Also, spirometry is a global total lung and airways assessment, which cannot differentiate a regional pattern of tissue destruction, and has limited ability to differentiate the type of compartments affected.

The present Research Topic aimed to present the current plethora of techniques in pulmonary functional research based on radiologic imaging, which are scaled from small animal models to clinical imaging in patients with lung disease. The chain of quantification ranges from inherently quantitative techniques such as T1 mapping with magnetic resonance imaging (MRI) to delicate post-processing encompassing segmentation of lungs, vessels, airways and pulmonary abnormalities with heuristic and, more recently, artificial intelligence-based methods, and very often combinations of these. At the same time, the original works on display in this Research Topic demonstrate the strong translational focus of our field of research.

The works by Almeida et al., Ji et al., and Konietzke et al. sought to capture structural and functional abnormalities in the chronic obstructive pulmonary disease (COPD) lung by quantitative post processing. Ji et al. separated COPD patients into three groups based on the visual impression of interstitial lung abnormalities (ILA) accompanying the typical imaging features of COPD (1). The authors could show that in the group of COPD patients with visual presence of ILA, airway wall thickness was increased. Konietzke et al. performed a thorough analysis of airway wall and parenchymal metrics using the GOLD stage, demonstrating that emphysema severity incrementally increases with every GOLD stage whereas airway wall thickening stagnated from GOLD II (2). Almeida et al. complemented these metrics with a novel artificial intelligence-based self-supervising network to detect anomalies in lung structure in COPD, adding to the traditional measurement of lung density to detect emphysema. The authors demonstrated good correlations of this AI-based abnormality score with traditional CT metrics and lung function decline. A novel but increasingly studied CT technique based on phase contrast (rather than absorption alone) was applied by Dullin et al. to a mouse model of allergic airway disease employing a synchrotron-derived x-ray source. The study by Yoshida et al. used a very large x-ray dataset in conjunction with a deep learning network to successfully predict spirometry values. Though the actual mechanism through which AI can derive such information remains obscure, it is a strong demonstration of how well imaging data is connected to lung function.

Magnetic resonance imaging (MRI) holds great potential for repeat and functional assessments of the lung, making it an ideal research tool. But it has also been introduced into clinical routine for a number of specific indications such as cystic fibrosis, primary ciliary dyskinesia and Pancoast tumor (3, 4). The works by Doellinger et al., Mummy et al., Ringwald et al., and Triphan et al. dive deep into this dynamic research field of functional lung MRI, using three different technological approaches. Mummy et al. explored the impact of anthropomorphic details such as age, sex and body mass index on MRI-derived measures of pulmonary gas exchange with hyperpolarized ^129^Xe-MRI. Since this technique holds great potential to regionally assess impairments in the air-to-blood diffusion barrier, such work is of great importance for implementing this advanced technique in clinical routine at specialized centers (5). Doellinger et al. used a completely contrast agent-free approach to assess pulmonary ventilation and perfusion solely from time-resolved acquisitions in combination with registration and a matric-pencil decomposition, which separates cyclic changes of MRI signal intensity into contributions of pulsatile blood inflow and respiration (6). The authors could demonstrate a good correlation with the more widely established contrast-enhanced 4D perfusion technique and score, opening a perspective for contrast-free assessments of cystic fibrosis lung disease. Ringwald et al. used a large MRI dataset incl. 4D perfusion data to train a convolutional neural network to segment the lungs in children with cystic fibrosis. This marks progress in the effort to make MRI evaluation less user-dependent. Triphan et al. used another approach by directly quantifying the T1-relaxation times in an echo-time dependent manner in COPD patients. Apparently, shorter T1 at ultra-short echo times is more correlated with tissue abnormalities, whereas at longer echo times it is more correlated with perfusion (7). Thus, this technique allows for a sub-resolution assessment of tissue composition.

Finally, the systematic review by Hofmann et al. on the effects of vaping on the lungs is a nice summary of the different techniques used (CT, MRI and FDG-PET/CT) to study the structural and functional impairment caused by inhalative toxins.
